# Clinical skills of general practitioners in Nairobi, Kenya: a cross-sectional study

**DOI:** 10.3399/BJGPO.2021.0233

**Published:** 2022-06-29

**Authors:** Gulnaz Mohamoud, Robert Mash

**Affiliations:** 1 Division of Family Medicine and Primary Care, Stellenbosch University, Stellenbosch, South Africa; 2 Department of Family Medicine, Aga Khan University, Nairobi, Kenya

**Keywords:** primary health care, general practice, family medicine, clinical skills, comprehensive health care, private sector, Primary care

## Abstract

**Background:**

Quality service delivery in primary care requires motivated and competent health professionals. In the Kenyan private sector, GPs with no postgraduate training in family medicine offer primary care. There is a paucity of evidence on the ability of primary care providers to deliver comprehensive care and no such evidence is available for GPs practising in the private sector in Kenya.

**Aim:**

To evaluate GPs’ training and experience in the skills required for comprehensive primary care.

**Design and setting:**

A cross-sectional descriptive survey in 13 primary care clinics in the private sector of Nairobi, Kenya.

**Method:**

A questionnaire, originally designed for a national survey of primary care doctors in South Africa, was adapted. The study collected self-reported data on performance of clinical skills by 25 GPs. Data were analysed using the Statistical Package for Social Sciences (SPSS, version 25).

**Results:**

GPs were mostly aged <40 years, with ≤10 years of experience, and there was an equal sex distribution. GPs reported moderate performance with adult health, communication and consultation, and clinical administration; and weak performance with emergencies, child health, surgery, ear, nose, and throat (ENT) and eyes, women’s health, and orthopaedics. The GPs lacked training in specific skills such as proctoscopies, contraceptive devices, skin procedures, intra-articular injections, red reflex tests, and use of genograms.

**Conclusion:**

GPs lacked training and performed poorly in some of the essential skills required in primary care. Continuing professional development, training in family medicine, broadening the model of care, and deployment of family physicians to the clinics could improve care comprehensiveness.

## How this fits in

High-quality primary care means delivering comprehensive care services across a person’s life course, which helps to reduce the burden of disease and ranges from health promotion to palliative care. GPs should be competent in a range of clinical skills that support comprehensiveness. This study finds that GPs in the Kenyan private sector are not able to offer a comprehensive service. These mostly young doctors, without postgraduate training in family medicine, might benefit from continuing professional development, support from family physicians, and changes to the model of service delivery to provide a more comprehensive service.

## Introduction

The signing of the Declaration of Astana reaffirmed the commitment of the World Health Organization (WHO) and member states to *’prioritise, promote and protect people’s health and wellbeing and provide health services that are of high quality, safe, comprehensive, integrated, accessible, available, and affordable for all‘*.^1,2^ To achieve such goals, health systems have to be built on the foundation of primary health care.^3,4^ Primary health care has been defined as having three key levers, namely, primary care, multi-sectoral action, and community empowerment.^5^ Primary care in sub-Saharan Africa (SSA) faces difficulties such as hospital-centred health priorities, fragmentation of health care in vertical programmes, dual burdens of communicable and non-communicable diseases, as well as healthcare workers who are not well trained.^1,6,7^

Primary care is the first contact with patients and a gatekeeper to other levels of health care.^8–10^ However, quality primary care requires accessible, coordinated, and ongoing comprehensive care.^11^ Primary care may be delivered by family physicians, GPs, or non-physician practitioners such as nurses.^8^ Comprehensive care should be offered across the life course, aim to reduce burden of disease, and should range from health promotion to palliative care.^11^ Comprehensive services depend on the availability of essential medicines, medical equipment, supplies, appropriate infrastructure, adequate funding, functional health information systems, and an available and competent workforce.^11–13^

In Kenya, primary care providers include family physicians, GPs, clinical officers, and nurses. Family physicians are doctors with 4 years of postgraduate training to become specialists in family medicine. GPs are qualified doctors who work in primary care without any postgraduate qualifications in family medicine and work mostly in the private sector. Clinical officers are mid-level doctors who, together with nurse practitioners, work mostly in the public sector.^14^ There is a significant gap between the competency of GPs with no postgraduate training and the competencies required of primary care providers.^15^ Specialist training in family medicine can reduce this competency gap,^3,16,17^ but the availability of family physicians in Kenya, as in most of SSA, is very limited.^18^ Therefore, additional in-service training and clinical skills enhancement of GPs, such as that outlined in the South African Diploma in Family Medicine,^19^ may contribute significantly towards the achievement of quality primary care.^20^

The measurement of primary care performance guides the strengthening of primary health care, which is a vital part of the health system.^11,21^ Nevertheless, in SSA there is a dearth of information on the quality of service delivery and performance of providers in primary care.^11,21,22^ Governments focus their attention on public sector services and measurement of primary care quality in the private sector is even more scarce.^23^ The proportion of the services provided by private organisations (56%) in Kenya is increasing; however, the quality of services remains a major challenge.^24^ A knowledge gap exists in the Kenyan private sector on the ability of GPs to deliver quality primary care. Therefore, the aim of this study was to evaluate the training and experience of GPs in the clinical skills required for comprehensive primary care.

## Method

### Study design

This was a cross-sectional descriptive survey using an adapted South African questionnaire.^25^

### Setting

The study was conducted in 13 primary care facilities within the city of Nairobi, which were run by GPs. These facilities were attached to a not-for-profit private healthcare organisation that also operated an associated tertiary hospital. The majority of patients were covered by health insurance. The facilities offered services to all age groups in semi-urban, urban, and peri-urban areas of Nairobi. The facilities provided health promotion, disease prevention and treatment services, and referred patients to specialist clinics (including family medicine), and offered rehabilitative and palliative care at the tertiary hospital. There were 25 GPs working in these facilities on a shift basis, according to the workload and opening schedules.

### Study population and sampling strategy

The study included all 25 GPs working in the 13 primary care facilities, without sampling, and there were no exclusion criteria.

### Data collection

The questionnaire was developed in South Africa for a national survey of primary care doctors in both public and private sectors. The selection of 78 clinical skills was based on the primary care subset of clinical skills required in the training of family physicians.^25^ The skills were divided into the following categories: adult health; women’s health; child health; surgery; orthopaedics; emergencies; ENT and eyes; clinical administration; and communication and consultation. Demographic data, such as age, sex, years of experience, and additional qualifications, were also collected. The GPs assessed their ability in performing each clinical skill by choosing one answer from a four-point Likert scale:

1. I have not had training in this skill.
2. I have been trained, but have not performed this skill in the last year.
3. I have performed this skill in the last year.
4. I have taught this skill to others in the last year.

The principal investigator, two Kenyan family physicians, and two GPs reviewed the relevance and appropriateness of the skills for the Kenyan private sector context. Skills related to circumcision, Norplant insertion, Rinne's and Weber’s tests, and ophthalmoscopy were added, while skills related to cricothyroidotomy, assessment of drunk driving, and termination of pregnancy were removed. Clinical administration skills in the form was adjusted to the Kenyan context.

The revised tool was then assessed for feasibility and understanding through a pilot study among GPs at a similar primary care facility that was not included in the study.

The GPs completed the paper-based questionnaires, which were collected by one researcher who ensured that they were fully completed.

### Data analysis

The data were captured in a Microsoft Excel spreadsheet and imported into the SPSS (version 25) to be analysed by the principal researcher. A mean score for each category of skills was created, based on the Likert scale from 1–4.

The data were analysed descriptively using frequency counts and percentages for categorical data. A mean score, based on the Likert scale from 1–4, was also calculated for each clinical skill. The mean scores were further categorised into poor performance (1.0–2.0: lack of training and not performed recently), weak performance (2.1–2.5: trained, but not performed recently), moderate performance (2.6–3.0: mostly performed in the last year), and strong performance (>3.0: performed often and taught to others).

The scores for each category were reported as medians and interquartile ranges (IQRs) as they were not normally distributed. Sex was compared with the median score per skill category using the non-parametric independent samples median test. Age and years of experience as numerical variables were correlated with the median score per skill category using the non-parametric Spearman’s correlation.

## Results

All of the GPs agreed to participate and Table 1 shows their sociodemographic characteristics. They were mostly aged <40 years, with ≤10 years of experience, and there was an almost equal sex distribution. Out of 25 GPs, two had additional non-clinical qualifications at a master’s level and five were postgraduate students in other disciplines.

**Table 1.** Sociodemographic characteristics of the GPs (*n* = 25)

Variable	*n* (%)
**Sex**	
Female	13 (52)
Male	12 (48)
**Age, years**	
20–29	6 (24)
30–39	15 (60)
40–49	2 (8)
50–59	2 (8)
**Years since qualification**	
1–10	20 (80)
11–20	3 (12)
21–30	1 (4)
31–40	1 (4)

Table 2 shows the median (IQR) scores of GPs across the clinical skills categories. Categories with an overall moderate performance included adult health, communication and consultation skills, and clinical administration. The categories with weak performance included emergencies, child health, surgery, ENT and eyes, women’s health, and orthopaedics.

**Table 2.** Performance of key clinical skills by categories (*n* = 25)

Categories	Median (IQR)
Adult health	2.8 (2.5–3.3)
Emergencies	2.5 (2.3–3.6)
Communication and consultation skills	2.7 (2.4–2.9)
Child health	2.5 (2.3–2.9)
Clinical administration skills	2.7 (2.3–3.0)
Surgery	2.4 (2.0–3.0)
Ear, nose, and throat and eyes	2.4 (2.1–2.7)
Women’s health	2.3 (2.0–2.5)
Orthopaedics	2.1 (1.6–2.4)

IQR = interquartile range.

Table 3 shows the relationship between sex and performance of skills in different categories. There were no differences between the sexes, apart from a significantly higher median score for males in relation to adult health (3.0 versus 2.6; *P* = 0.047).

**Table 3.** Relationship between sex and self-reported performance of skills by category

Category	Male,*n* = **12,**median score (95% CI)	Female,*n* = **13,**median score (95% CI)	*P* value
Adult health	3.0 (2.5 to 3.5)	2.6 (2.5 to 2.8)	0.047
Emergencies	2.7 (2.4 to 3.6)	2.5 (2.3 to 3.1)	0.434
Communication and consultation skills	2.7 (2.3 to 3.3)	2.7 (2.7 to 2.9)	0.695
Child health	2.5 (2.3 to 3.3)	2.6 (2.3 to 2.9)	0.695
Clinical administration skills	3.0 (3.0 to 4.0)	2.3 (2.3 to 3.0)	0.111
Surgery	2.6 (2.3 to 3.2)	2.2 (1.9 to 2.5)	0.115
Ear, nose, and throat and eyes	2.6 (2.4 to 3.0)	2.3 (1.9 to 2.6)	0.434
Women’s health	2.3 (2.1 to 2.8)	2.3 (1.9 to 2.5)	0.999
Orthopaedics	2.4 (2.0 to 2.6)	1.9 (1.6 to 2.4)	0.115

Table 4 shows the relationship between age or years of experience and performance of skills in different categories. For most categories, there were no significant relationships with age or years of experience. However, clinical administrative skills improved significantly with both age (*r* = 0.492; *P* = 0.012) and years of experience (*r* = 0.513; *P* = 0.009), and communication and consultation skills with years of experience (*r* = 0.411; *P* = 0.041).

**Table 4.** Relationship between age and years of experience and self-reported performance of skills by category

Category	Correlation coefficient (*r*)	*P* value
**Correlation between age in years and score for:**		
Adult health	0.254	0.220
Emergencies	–0.028	0.894
Communication and consultation skills	0.372	0.067
Child health	0.001	0.996
Clinical administration skills	0.492	0.012
Surgery	0.260	0.210
Ear, nose, and throat and eyes	0.348	0.088
Women’s health	0.021	0.921
Orthopaedics	0.174	0.406
**Correlation between years of experience and score for:**		
Adult health	0.283	0.171
Emergencies	–0.028	0.895
Communication and consultation skills	0.411	0.041
Child health	–0.053	0.801
Clinical administration skills	0.513	0.009
Surgery	0.247	0.234
Ear, nose, and throat and eyes	0.358	0.079
Women’s health	0.167	0.425
Orthopaedics	0.217	0.298

Supplementary Table S1 presents the mean score for each skill and Supplementary Table S2 categorises the clinical skills according to their mean scores into poor, weak, moderate, and strong performance. GPs scored poorly in 10 (13%) skills, weakly in 23 (29%), moderately in 26 (33%), and strongly in 19 (24%). The GPs lacked training in specific skills related to proctoscopies, contraceptive devices, skin procedures, intra-articular injections, red reflex tests, and use of genograms.

## Discussion

### Summary

The majority of the GPs were young doctors with ≤10 years of clinical experience and some were specialising in other disciplines. The GPs performed moderately in the categories of adult health, clinical administration, and communication and consultation skills. Male GPs reported better performance in skills related to adult health. Administrative and communication skills also improved significantly with age and years of experience. The GPs performed weakly in the categories of child health, emergencies, surgery, women’s health, ENT and eyes, and orthopaedics. Their strongest self-rated performance for individual skills were interpreting X-rays, suturing lacerations, inserting urinary catheters, performing patient-centred consultations, and HIV counselling. Their poorest performance for individual skills was seen in their ability to draw genograms, give intra-articular injections, perform family planning procedures, perform biopsies, conduct proctoscopies, give red reflex tests, and perform obstetric ultrasound.

### Strengths and limitations

To the authors’ knowledge, this study is the first of its kind to evaluate GPs’ performance in the private sector in Kenya; however, the study has limitations. Data regarding the GPs’ undergraduate training were not captured and this could have been a useful factor to consider. Since the scores were self-reported, the GPs may have overestimated their performance. Scoring was largely based on prior training and whether the skills had been performed, and thus could not fully assess competence. The questionnaire focused more on the frequency with which a skill was performed rather than the quality of performance. Competence can only be reliably assessed by direct observation of skills.

The results are representative of GPs practising in this specific chain of primary care clinics and the results cannot be generalised to GPs working elsewhere in the private sector in Kenya. However, it is likely that similar findings would be obtained for GPs working in similar private sector organisations in urban settings and the recommendations could have relevance to them.

### Comparison with existing literature

The GPs reported strong performance in certain emergency skills, such as the Glasgow Coma Scale, inserting urinary catheters, administering oxygen, and carrying out primary and secondary surveys, although these are not common in general practice.^25^ The organisation, which also managed the tertiary hospital, required all doctors to be accredited in advanced life support. In addition, several GPs were registrars in other disciplines and may have performed emergency skills in those contexts.

The GPs’ high scores in interpreting electrocardiograms and radiographs may have been owing to their relatively recent hospital-based internship training, where they were required to interpret the findings for themselves. The COVID-19 pandemic may have also contributed to the increased number of chest radiographs performed during the study.^26^

GPs reported strong performance in consultation and communication skills, although communication skills are challenging to define and self-assess.^27^ Indirect observation of the GPs’ consultations in a previous study carried out at these facilities showed that consultations were brief, biomedical, and lacked a person-centred approach.^28^ Paradoxically, those who are most confident about their communication skills can be the least competent when independently assessed.^27^

There were skills that the GPs in this study were trained in, but had not performed in the past year, such as medical circumcisions, fine-needle aspiration biopsies, excision of sebaceous cysts, and aspects related to sexual and child abuse. In South Africa, GPs reported high confidence in surgical skills and this may be because the study included rural areas where specialists were less accessible.^29,30^ In addition, the clinics may not have provided all the equipment needed for these procedures and GPs become deskilled over time when procedures are not performed frequently.^30^ Patients also had limited expectations of the scope of practice at these primary care facilities and may have bypassed primary care to attend the tertiary hospital.^31^

Specialist services were easily available at the tertiary hospital and covered by medical insurance. Therefore, it may have been easier for the GPs to refer patients to a specialist and not perform the skill themselves.^32^ It has been reported that easy availability of specialists can narrow the scope of practice of urban GPs.^30^ Reliance on specialists could also be related to fear of litigation in the private sector, which has risen in recent years.^33,34^

Remuneration of GPs was organised per session, which meant there was no financial incentive to do more than the minimum required. On the other hand, most of these young doctors were not pursuing a career in family medicine and saw this as a source of additional income, while they trained for another specialty. Hence, their motivation to perform skills attuned to general practice may have been low. It is also possible that the COVID-19 pandemic may have contributed to the withdrawal of some services that were considered to be high risk. However, despite these gaps, the overall satisfaction with the GPs was very high, as expressed by the patients in a different study carried out at these clinics.^20^

Some of the weak and poorly performed skills were basic primary care skills, such as use of Snellen charts, genograms, family planning procedures, proctoscopies, hearing tests, or use of peak flow metres. This may be an indication that the GPs were not adequately prepared for primary care during undergraduate training or as junior doctors in the hospital environment. In addition, lack of equipment and reliance on specialist could also be a contributory factor.

### Implications for research and practice

This study points towards a number of ways in which the comprehensiveness of care could be increased and improved. Perhaps shifting the focus of the Department of Family Medicine from the tertiary hospital to primary care and employing new family medicine graduates could ensure that family physicians take the lead in these clinics. Family physicians with their postgraduate training would immediately increase the scope and quality of care, and create a career pathway for doctors that want to work in primary care.^18,35^ Family physicians could also introduce a culture of clinical governance, with attention given to improving the quality of care and patient safety.^36^

Continuing professional development and workplace-based experiential learning^37^ for GPs under the supervision of family physicians would also help to address the skills gap. More formal training similar to the South African Diploma in Family Medicine could also upgrade the GPs and bridge this gap.^18,25,32,38^

Attention should be given to the design of the health system and financing mechanisms. The population served by this private sector organisation had developed low expectations of the scope of practice in the primary care clinics,^31^ even though they were satisfied with the services they received.^20^ A redesign of the health system’s approach to gatekeeping and care pathways could encourage patients to obtain more cost-effective and accessible services at the primary care clinics. An important factor to consider would be incentives, such as fee-for-service, to motivate GPs to perform some of the procedures such as circumcision.^32^ A change in the scope of practice would also need to be supported by the organisation’s infrastructure, equipment, supply chain, workforce, funding, and health information systems.

Studies in primary care settings in the public sector would provide comparative results. Qualitative studies may also be useful to explore the factors influencing GPs’ clinical skills in more depth. Kenya is one of the ‘trailblazer countries‘ for the Primary Health Care Performance Initiative and yet has little data to measure its performance.^39^ Further research that measures the quality of service delivery in terms of access, coordination, continuity, person-centredness, and comprehensiveness is needed. The Primary Care Assessment Tool was recently adapted for use in Kenya and this may be an important tool to assist with this measurement.^28^

In conclusion, the majority of the GPs were young and not considering a career in family medicine. There was moderate performance of skills in the categories of adult health, clinical administration, and communication and consultations. There was weak performance of skills in the categories of child health, emergencies, surgery, women’s health, ENT and eyes, and orthopaedics. GPs performed poorly in some of the essential and basic skills required in primary care. Comprehensiveness could be improved by deploying family physicians to the clinics, continuing professional development and postgraduate training, as well as re-engineering of the health system and financing mechanisms.
